# Supplementary figures and images for: Online college English education in Wuhan against the COVID-19 pandemic: Student and teacher readiness, challenges and implications
Source: PLoS One. 2021 Oct 1;16(10):e0258137. doi: 10.1371/journal.pone.0258137 (PMC8486088; doi:10.1371/journal.pone.0258137)

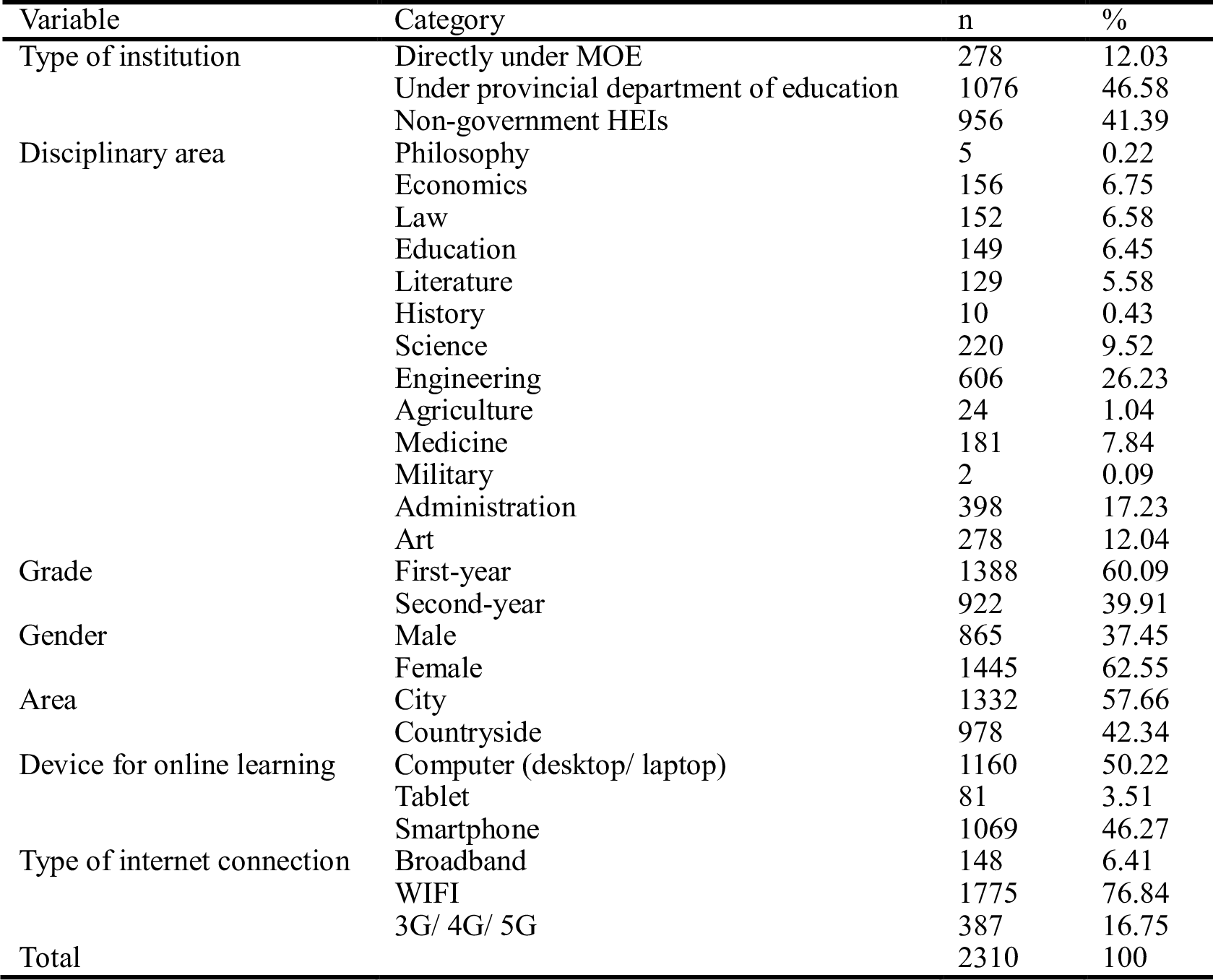

Supplement: S1 Table — (TIF) [file pone.0258137.s001.tif]

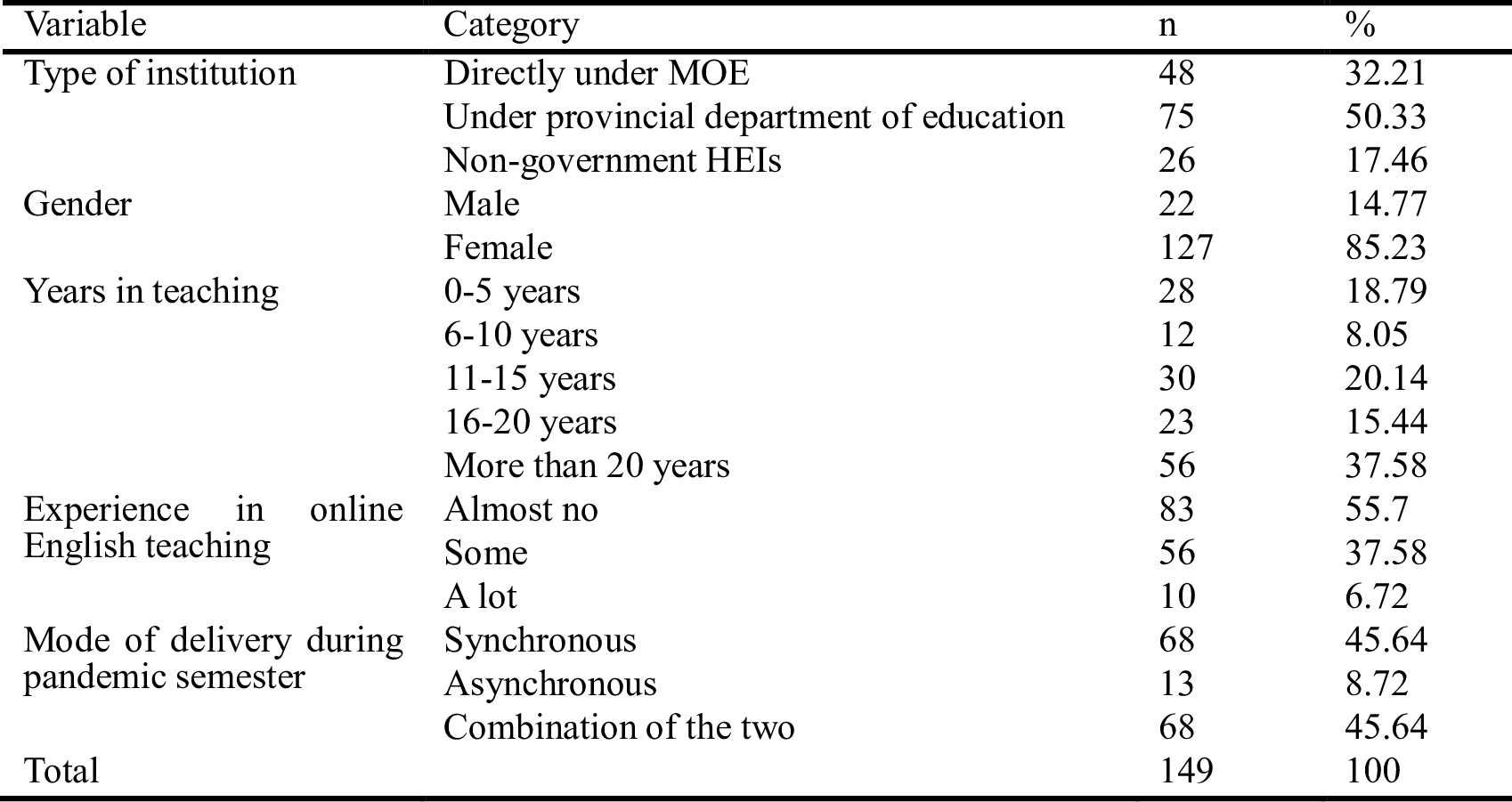

Supplement: S2 Table — (TIF) [file pone.0258137.s002.tif]

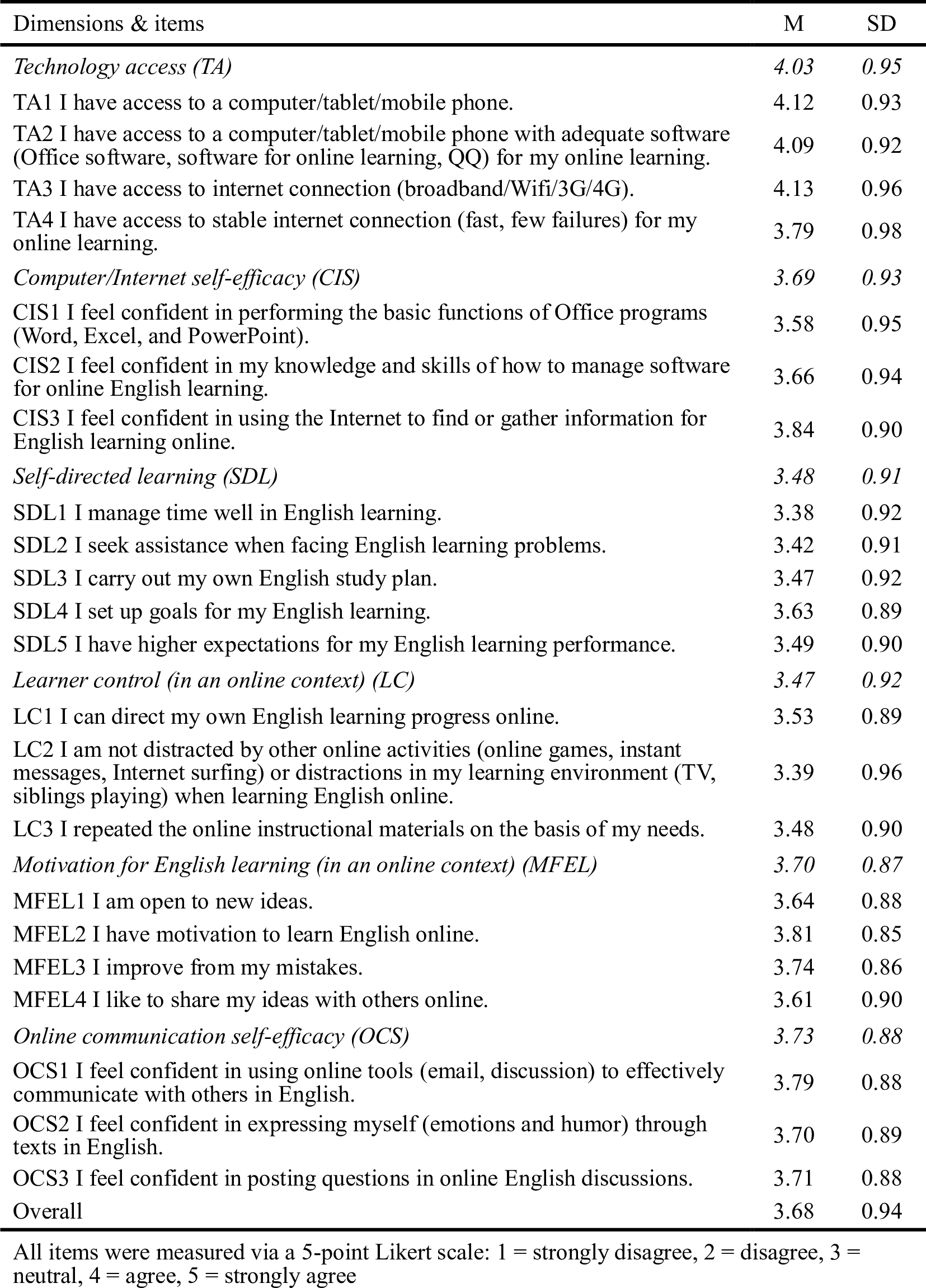

Supplement: S3 Table — (TIF) [file pone.0258137.s003.tif]

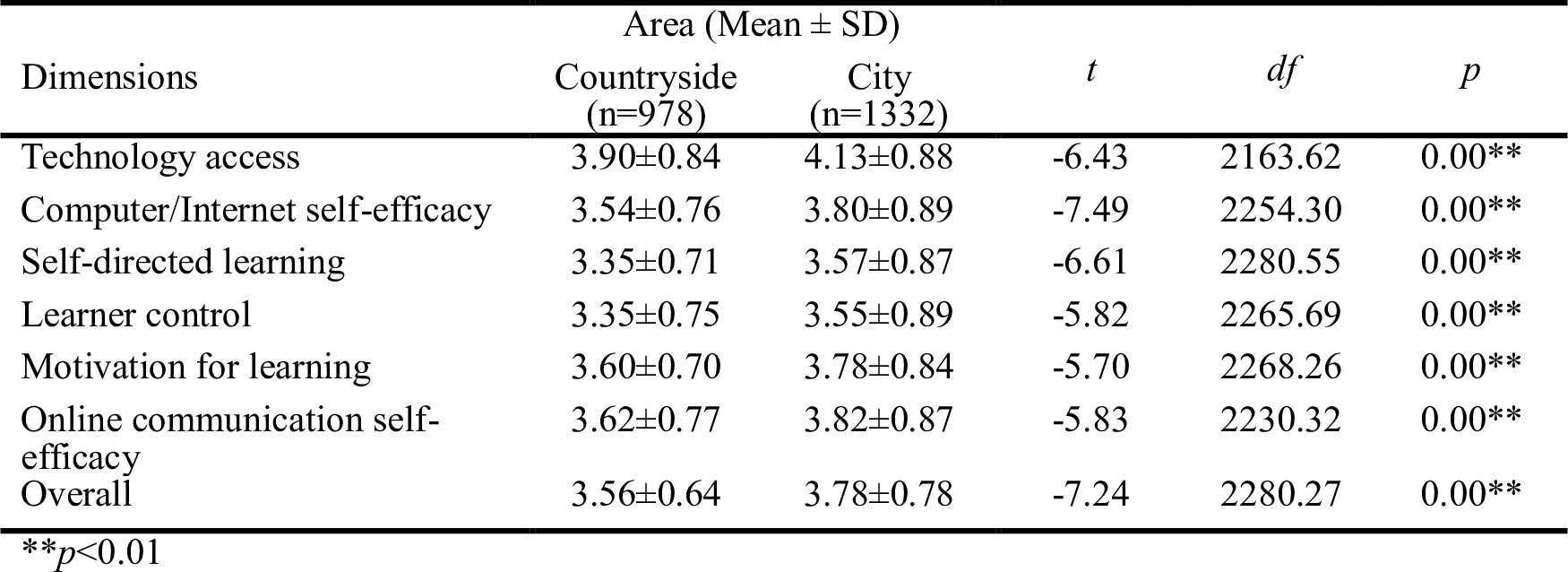

Supplement: S4 Table — (TIF) [file pone.0258137.s004.tif]

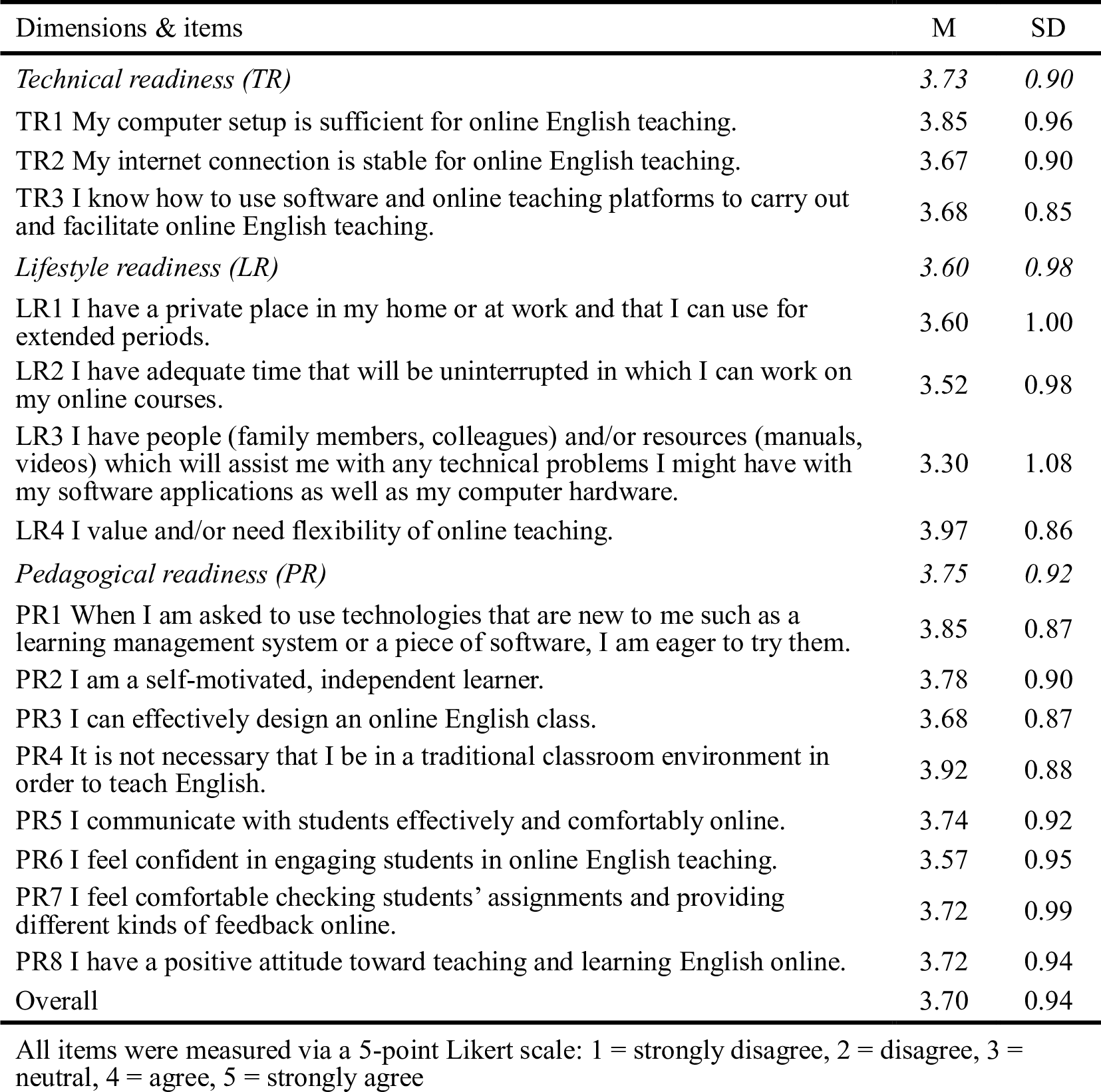

Supplement: S5 Table — (TIF) [file pone.0258137.s005.tif]

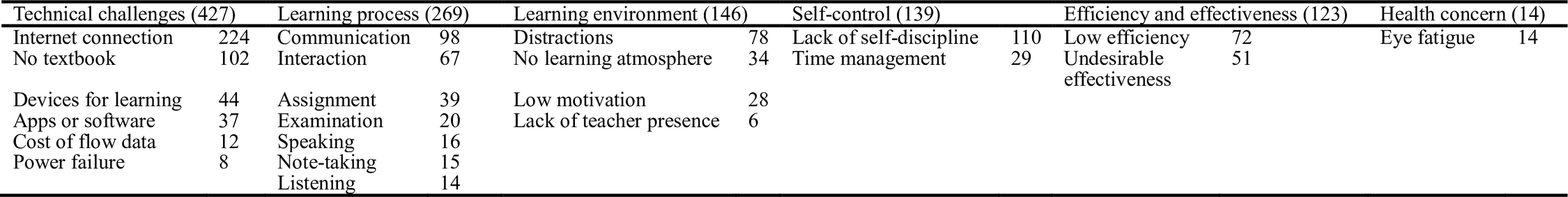

Supplement: S6 Table — (TIF) [file pone.0258137.s006.tif]

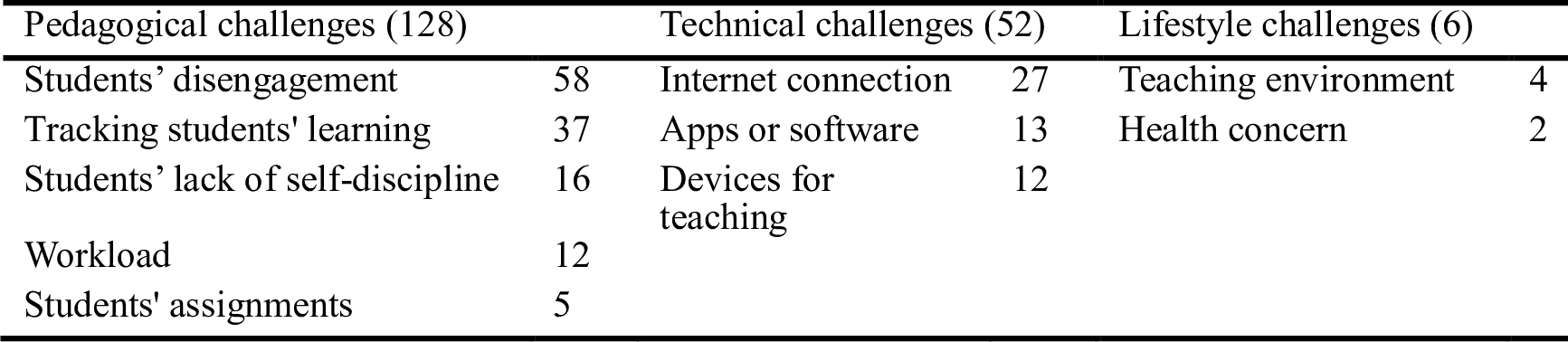

Supplement: S7 Table — (TIF) [file pone.0258137.s007.tif]

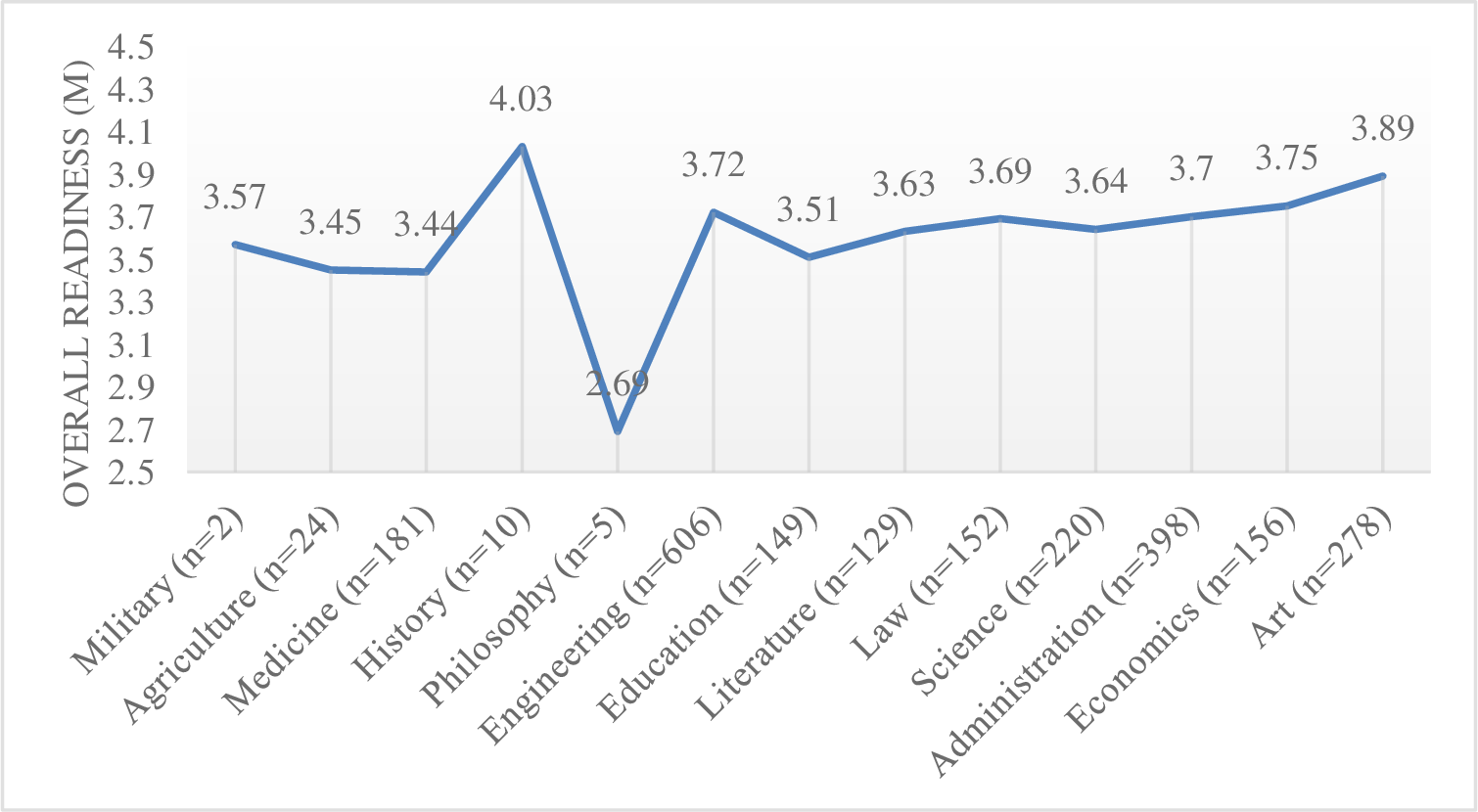

Supplement: S1 Fig — (TIF) [file pone.0258137.s008.tif]
