# Supplementary material for: Online college English education in Wuhan against the COVID-19 pandemic: Student and teacher readiness, challenges and implications
Source: PLoS One. 2021 Oct 1;16(10):e0258137. doi: 10.1371/journal.pone.0258137 (PMC8486088; doi:10.1371/journal.pone.0258137)
Supplement: S1 File — (ZIP) [file pone.0258137.s009.zip › Questionnaire and interview results/Interview results.docx]

**Institution directly under MOE 1：**

1. 贵校疫情之前大学英语是否已采取在线或者混合教学的模式？（如果是，请简单介绍一下）

疫情前开展了混合式教学改革，学校有若干自建慕课。

2. 疫情期贵校的大学英语课主要采取什么教学模式（直播/现有慕课/临时录课等）？为什么这么安排呢？

大学英语主要是配合慕课资源，实施直播教学，少数录播；主要采用微师，QQ屏幕分享，微助教，雨课堂等。

3. 疫情期大学英语教学过程中是否使用了其它教学平台，比如出版社提供的教材配套学习平台？

使用了出版社配套电子教材WELEARN，U校园，批改网。

4. 本学期大学英语课程期末考核是如何进行的？较之前的传统考核方式有哪些改变？

加大平时考核比重至70%（多样化的平时作业，出勤，课堂互动，U校园听力考试），期末考核30%（思辨写作，小组合作项目）。

5. 将来贵校的大学英语教学是否会朝着网络教学的方向改革？为什么？

大学英语教学会根据需要和当时的条件，在学校许可的情况下，开展各种教学，如线上教学，线下教学，线上线下同步或异步教学等。采用SPOC，SPOC+PAD等教学模式。

**Institution directly under MOE 2：**

1. 贵校疫情之前大学英语是否已采取在线或者混合教学的模式？（如果是，请简单介绍一下）

疫情前以线上课堂面授为主，课后线上自主学习为辅，部分老师进行了混合教学模式，并进行慕课录制。

2. 疫情期贵校的大学英语课主要采取什么教学模式（直播/现有慕课/临时录课等）？为什么这么安排呢？

疫情期间都是直播课，老师们上直播课都是根据自身网络状况选择一些平台，比如腾讯课堂，腾讯会议，ZOOM等，主要是在完成教学任务，保证教学质量的前提下尽可能不给师生添加太多额外的压力。

3. 疫情期大学英语教学过程中是否使用了其它教学平台，比如出版社提供的教材配套学习平台？

使用了教材配套的学习平台，让学生进行课后自主学习。

4. 本学期大学英语课程期末考核是如何进行的？较之前的传统考核方式有哪些改变？

本学期期末考核并没进行，移到下学期开学进行线下考试。

5. 将来贵校的大学英语教学是否会朝着网络教学的方向改革？为什么？

大学英语教学不会朝全网络教学方向改革，毕竟课堂上现实中的师生课堂互动更有效，学生实际也不大喜欢全网络教学，但是会进一步深化混合式课堂的教学改革探索。

**Institution directly under MOE 3：**

1. 贵校疫情之前大学英语是否已采取在线或者混合教学的模式？（如果是，请简单介绍一下）

是的。如:《大学英语A3》、《西方文化经典导读》、《高级英语听力》等大学英语课程在疫情之前已立项校级在线课程，开展混合式教学。

2. 疫情期贵校的大学英语课主要采取什么教学模式（直播/现有慕课/临时录课等）？为什么这么安排呢？

主要采取直播、现有慕课或学习平台相结合的教学模式。既能保证在课堂上师生互动交流 同时课下学生也可以通过现有慕课平台或学习平台进行自主学习并完成作业。

3. 疫情期大学英语教学过程中是否使用了其它教学平台，比如出版社提供的教材配套学习平台？

是的，疫情期大学英语新生年级使用了出版社（外研社、清华社）教材配套学习平台。

4. 本学期大学英语课程期末考核是如何进行的？较之前的传统考核方式有哪些改变？

18级大学英语期末考试采取论文或大作业形式进行（已完成），与以往主要采取闭卷考试的考核方式不同；19级大学英语期末考试学生秋季返校后进行（考核方式未发生改变）。

5. 将来贵校的大学英语教学是否会朝着网络教学的方向改革？为什么？

本校大学英语教学会采取线下教学为主、线上教学为辅的混合式教学模式，拟实现线上教学资源与线下个性教学的深度融合，充分发挥各自的优势。

**Institution under provincial department of education 1：**

1. 贵校疫情之前大学英语是否已采取在线或者混合教学的模式？（如果是，请简单介绍一下）

之前我们采用了朗文交互英语课程，这一课程为基于多终端的学生在线自主学习平台，以听说读写训练为主。作为课堂教学的补充。

2. 疫情期贵校的大学英语课主要采取什么教学模式（直播/现有慕课/临时录课等）？为什么这么安排呢？

疫情期间教学：自建在线课程+直播模式。考虑到学生在家没有课本，我们自建了在线课程，其中纳入了课程教学知识点。直播是为了通过练习、讲解等互动手段让学生更好地掌握知识点，厘清重难点。

3. 疫情期大学英语教学过程中是否使用了其它教学平台，比如出版社提供的教材配套学习平台？

利用了优学院平台实现课堂管理，如考勤、作业等。

4. 本学期大学英语课程期末考核是如何进行的？较之前的传统考核方式有哪些改变？

较之以前的评价方式，这学期考评的变化（1）提高了形成性评价在总评中的比例，由40%提高到70%，以前总评成绩形成性评价占40%，终结性评价60%（具体包括：在线朗文交互英语20%，课堂表现20%，一对一口语考试10%，期末笔试50%）；这学期形成性评价占70%，（包括在线朗文交互英语课程20%，在线课件学习20%，课堂直播表现30%），终结性评价占30%（一对一口语考试15%，期末笔试15%）。（2）之前期末笔试题型包括听力、单项选择、翻译，阅读及写作，既包括主观题也包括客观题。本学期期末考试包括阅读理解及基于阅读的写作，均为主观题。（3）以前期末笔试集中考试，本学期利用优学院平台在线进行。

5. 将来贵校的大学英语教学是否会朝着网络教学的方向改革？为什么？

未来会将网络教学纳入教学体系。基于网络的课程教学更有利于学生个性化的学习。另，教师们经过一个学期的在线教学，对在线教学已较为熟悉。

**Institution under provincial department of education 2：**

1. 贵校疫情之前大学英语是否已采取在线或者混合教学的模式？（如果是，请简单介绍一下）

疫情之前已经开始采用混合式教学模式尝试，主要是利用U校园教学平台和线下课堂教学相结合的教学方式。U校园教学平台的主要作用为提供课前预习资源，课堂或课后检测，课外资源补充发布，课后练习、复习，辅助学生自主学习等等。由于U校园平台的课堂互动功能不足，课堂教学一般采用雨课堂或者超星学习通为课堂互动平台，记录学生课堂表现。

2. 疫情期贵校的大学英语课主要采取什么教学模式（直播/现有慕课/临时录课等）？为什么这么安排呢？

疫情期间大学英语课程根据各类课程性质的不同采用了多种教学模式。

大学英语类基础课程，如大学基础英语和英语技能训练课程主要采用U校园学习平台和直播、或者临时录课相结合的模式；学科英语类课程采取的是学习通平台共同建课、录课，QQ群或腾讯课堂定时答疑相结合的模式；英语语言拓展类课程采取现有慕课和QQ群或者腾讯课堂定时答疑相结合的模式。

由于疫情期间部分老师被困外地，手头教学资源、教学设备有限，因此采用了基于教学平台资源如U校园、慕课堂的，结合在线直播或者在线答疑等灵活的教学方法的教学模式。

学科英语类课程，由于线上资源有限，则采取了共同建课、录课，QQ群在线答疑的教学模式。

3. 疫情期大学英语教学过程中是否使用了其它教学平台，比如出版社提供的教材配套学习平台？

疫情期间主要的教学平台：U校园、中慕课、超星学习通，另外老师们还灵活使用了QQ群、微信群、腾讯课堂等平台进行教学。U校园是出版社提供的配套教学平台，因此大学英语类课程主要使用该平台。

4. 本学期大学英语课程期末考核是如何进行的？较之前的传统考核方式有哪些改变？

大学英语课程期末考核采用的都是线上考试形式。

考核难度、题型总体维持不变。由于考虑到学生在线答题的便利，以及在线答题的特殊性，如部分学生网络条件不好、部分学生打字速度较慢等，客观题的比例比往年有所提高，主观题比例下调。

5. 将来贵校的大学英语教学是否会朝着网络教学的方向改革？为什么？

目前大学英语课程的改革趋势为线上、线下相结合的混合式教学模式。部分课程，如英语语言拓展类课程，由于语言技能或者知识信息输入内容比较多，可以考虑开设线上课程。但部分课程如大学基础英语课程、学科英语类课程改为线上课程的条件还不成熟。

**Institution under provincial department of education 3：**

1. 贵校疫情之前大学英语是否已采取在线或者混合教学的模式？（如果是，请简单介绍一下）

采用混合式学习，使用学习通和U校园app进行在线教学

2. 疫情期贵校的大学英语课主要采取什么教学模式（直播/现有慕课/临时录课等）？为什么这么安排呢？

录课，直播都有。根据教师各自的网络条件而定。

3. 疫情期大学英语教学过程中是否使用了其它教学平台，比如出版社提供的教材配套学习平台？

学习通和U校园。

4. 本学期大学英语课程期末考核是如何进行的？较之前的传统考核方式有哪些改变？

学习通平台在线考试。主要是考试模式从线下移至线上，考核成绩比率不变。

5. 将来贵校的大学英语教学是否会朝着网络教学的方向改革？为什么？

不会。应该是线上和线下教学相结合，但还是以线上教学为主。

**Institution under provincial department of education 4：**

1. 贵校疫情之前大学英语是否已采取在线或者混合教学的模式？（如果是，请简单介绍一下）

我们学校一直在做混合教学，大一的通用英语，我们有网络学习平台作为辅助，而且对学生有要求，有些课程实际上网上自主学习完成，大二的我们有几门课程呢，最近从一七年开始都实行线上和线下相结合的混合教学模式，比如说英语畅谈中国，他已经是国家精品视频课程，英语演讲是湖北省的精品在线课程，去年申报了国家的估计会批下来，所以在线教学一直在实行。

2. 疫情期贵校的大学英语课主要采取什么教学模式（直播/现有慕课/临时录课等）？为什么这么安排呢？

大一的课程，我们主要是老师直播加学生线下完成网络学习平台的学习任务，这个学习平台主要是外研社和外教社的那个学习平台，大二的有慕课的呢，我们集合mooc进行教学，没有mooc的课程，主要是运用学习通线上直播QQ课堂等方式来进行线上教学。

3. 疫情期大学英语教学过程中是否使用了其它教学平台，比如出版社提供的教材配套学习平台？

我们使用教材出版社外研社和外教社提供的学习平台，还有慕课的平台，比如学堂在线等。

4. 本学期大学英语课程期末考核是如何进行的？较之前的传统考核方式有哪些改变？

这个学期的考核是比较特别的，我们以前的考核是四六开，平时成绩40%，期末考成绩60%，那么40%里面，有30%是考勤那作业，线上学习任务的完成情况，10%是那个期中考试，这个学期的大英一期末考核变成了七三，平时成绩是70%，考试成绩30%，我们的这个70%其中30%来自于综合教程30%，听说教程30%，然后10%是我们的那个比如说课堂参与，老师的考核考勤等。

我们大英二的考核有慕课的，稍微有点不一样，有mooc的课程在网络学习，这块儿看得更重，但是期末考试也只是占30%。期末考试是利用双平台在线考试，试卷编制和以往没有大的区别。
5. 将来贵校的大学英语教学是否会朝着网络教学的方向改革？为什么？

这个问题我不知道怎么看，就说你是说只搞网络教学还是线上线下相结合，如果说完全靠着网在线教学的方向，我估计应该不会，这个在线教学呢，只是一个临时的应急的一种方式，我个人觉得只能作为课堂教学的一种补充，比如说学习的内容的拓宽，自主学习能力的培养，然后阅读量的提高，可以通过线上的一些内容进行补充，弥补课堂教学的不足，比如说我们可以利用FiF口语训练系统去锻炼学生的语音，语调和口语，提升他们的口语水平，这是可以做到的，但是完全往在线教学方向发展不太可能，我个人觉得可能会线上线下相结合。比如说，利用学生现在的可以接触到的一些网络资源和学习平台，手机呀，电脑啊，可以给他们布置些网络学习任务，作为我们课堂教学的一些补充，我觉得这是可以的，就是线上线下相结合的混合式教学，应该是将来的发展方向。

**Institution under provincial department of education 5：**

1. 贵校疫情之前大学英语是否已采取在线或者混合教学的模式？（如果是，请简单介绍一下）

疫情前我校已采用混合教学模式，教师线下课堂教学和学生线上自主学习的形式，线上主要是自主完成听说读写相关练习和相关考试模拟测试，有学习平台自主平台自动记录学习成绩，本部分成绩计入期末成绩，占总评的20%。

2. 疫情期贵校的大学英语课主要采取什么教学模式（直播/现有慕课/临时录课等）？为什么这么安排呢？

疫情期根据教材选用采取直播结合他校现有慕课以及自建慕课的形式安排教学。

3. 疫情期大学英语教学过程中是否使用了其它教学平台，比如出版社提供的教材配套学习平台？

使用了出版社提供的教材配套学习平台。

4. 本学期大学英语课程期末考核是如何进行的？较之前的传统考核方式有哪些改变？

本学期期末考试移到下学期进行，线上自主学习的平时占比由20%增加到30%。

5. 将来贵校的大学英语教学是否会朝着网络教学的方向改革？为什么？

会朝着混合式教学模式的方向改革，因为通过这次实验，老师和学生其实很多人对在线教学还是比较积极的，正好我们学校也支持，所以我们会加大混合式教学模式改革的进度。

**Institution under provincial department of education 6：**

1. 贵校疫情之前大学英语是否已采取在线或者混合教学的模式？（如果是，请简单介绍一下）

之前有混合教学的模式，主要是学术英语。

2. 疫情期贵校的大学英语课主要采取什么教学模式（直播/现有慕课/临时录课等）？为什么这么安排呢？

直播、录播、现有在线课程均有，由老师自由选择，疫情能把课上下去就很不错啦，需要老师和他们班的学生共同商量决定。

3. 疫情期大学英语教学过程中是否使用了其它教学平台，比如出版社提供的教材配套学习平台？

听力用的U校园，就是教材配套的平台。

4. 本学期大学英语课程期末考核是如何进行的？较之前的传统考核方式有哪些改变？

我们采用在线开卷考试，考核题目都是主观题。跟以前不一样，以前是笔试闭卷，主客观都有。

5. 将来贵校的大学英语教学是否会朝着网络教学的方向改革？为什么？

混合式教学，一直是方向，大势所趋啊！

**Non-governmental institution 1：**

1. 贵校疫情之前大学英语是否已采取在线或者混合教学的模式？（如果是，请简单介绍一下）

疫情前并没有进行在线教学或者混合式教学。

2. 疫情期贵校的大学英语课主要采取什么教学模式（直播/现有慕课/临时录课等）？为什么这么安排呢？

疫情期间大学英语课主要采用直播方式进行。教学更好组织。

3. 疫情期大学英语教学过程中是否使用了其它教学平台，比如出版社提供的教材配套学习平台？

没有使用配套学习平台。

4. 本学期大学英语课程期末考核是如何进行的？较之前的传统考核方式有哪些改变？

期末考试安排6月底进行。双平台，每个学生打开摄像头，教师端监控，学生教学平台答卷，考核方式不变。

5. 将来贵校的大学英语教学是否会朝着网络教学的方向改革？为什么？

后期院里希望鼓励老师建设在线课程。跟进网络教学大趋势，更好跟进监督教学，提高教学质量。

**Non-governmental institution 2：**

1. 贵校疫情之前大学英语是否已采取在线或者混合教学的模式？（如果是，请简单介绍一下）
以前从未采用过在线或混合教学。
2. 疫情期贵校的大学英语课主要采取什么教学模式（直播/现有慕课/临时录课等）？为什么这么安排呢？
疫情期间，大学英语主要采用直播和录播课的形式。因为大学英语教材繁多，学生英语水平参差不齐，直播和录播结合有利于教师更好把握教学的进度和效果。
3. 疫情期大学英语教学过程中是否使用了其它教学平台，比如出版社提供的教材配套学习平台？
疫情期间，大学英语主要采用的教学平台有：腾讯会议、QQ、微信、钉钉等教学平台，出版社未提供配套的学习平台。
4. 本学期大学英语课程期末考核是如何进行的？较之前的传统考核方式有哪些改变？
期末考核以线上考核的形式进行，教师提前将答题纸发给学生打印，考试时教师将试卷发给学生，学生将答案写在答题纸上，拍照上交作为期末考试成绩，改变了传统的线下考试形式。
5. 将来贵校的大学英语教学是否会朝着网络教学的方向改革？为什么？
将来大学英语教学改革会考虑适当的网络教学，但仍以课堂教学为主！因为网络教学存在各种问题，在疫情期间暴露，完全的网络教学或者以网络教学为主，必须以学生高度的学习自觉性和主动性为前提，短期来说，我校学生很难达到这样的水平！

**Non-governmental institution 3：**

1. 贵校疫情之前大学英语是否已采取在线或者混合教学的模式？（如果是，请简单介绍一下）
疫情前并没有进行在线教学或者混合式教学。只有部分老师可能基于教改项目进行了一些混合式教学尝试，如微课，翻转课堂等，非常少。
2. 疫情期贵校的大学英语课主要采取什么教学模式（直播/现有慕课/临时录课等）？为什么这么安排呢？
疫情期间大学英语课主要采用直播方式进行。因为能确保课堂教学效果，了解学生对知识点掌握情况。
3. 疫情期大学英语教学过程中是否使用了其它教学平台，比如出版社提供的教材配套学习平台？
大学英语课使用了外研社学习平台。
4. 本学期大学英语课程期末考核是如何进行的？较之前的传统考核方式有哪些改变？
期末考试方式比率不变，但是变为了线上考试，通过摄像头监考。
5. 将来贵校的大学英语教学是否会朝着网络教学的方向改革？为什么？
可能后期部分老师会建设在线课程。他们建课可能是授课情况更能系统跟进，对学生学习情况统计更直观。
