# Supplementary material for: Online college English education in Wuhan against the COVID-19 pandemic: Student and teacher readiness, challenges and implications
Source: PLoS One. 2021 Oct 1;16(10):e0258137. doi: 10.1371/journal.pone.0258137 (PMC8486088; doi:10.1371/journal.pone.0258137)
Supplement: Attachment — Submitted filename: review results.docx [file pone.0258137.s010.docx]

Recommendation: Revisions Required

The paper investigated 2310 non-English-major students and 149 English teachers from three types of twelve higher education institutions in Wuhan, to evaluate their readiness for online English education, to figure out challenges they encountered, and to draw implications for future online college English education. It has various potentials and I recommend it to be considered for publication, but only after some major revisions. My suggestions are the following:

1. The authors failed to evaluate the context of the nation-wide online teaching in a justifiable way. They claimed that “This online teaching in the face of COVID-19, or exactly, online triage (Gacs et al. 2020), was carried out without need analysis or readiness evaluation from both learning and teaching sides and was different from well-prepared and planned online teaching.” But actually it was not the case: before the epidemic, online teaching had already serve as a positive supplement to offline teaching, and it is available and accessible for students in most part of China, both in rural and urban areas; there are many university websites and apps which offer free online courses, which quite a number of students make use of regularly in their spare time; great efforts have been made by education authorities, especially the Ministry of Education, to analysis and evaluate the situation, to invest considerably in providing online teaching resources, and to mobilize the education-related IT companies to give a helping hand; schools and teachers had tried their best to make preparations before the launch of the online courses. In short, this nationwide online teaching experience was not unprepared and unplanned at all. So, the authors should adjust their wording throughout the whole paper, in order not to make the basis of this research seemingly groundless.
2. Get the paper checked by expert speakers. The sentences are extremely long, which makes reading the text challenging. And occasionally, there are some inappropriate wording or even grammatical mistakes. All these may reduce the readability of this paper to some extent.
3. The teacher sample is relatively small, which makes the analysis and results less convincing.
4. The analysis would be more reasonable and sound, if the subjects of teachers and students were divided into two groups, rural and urban.

5. It would be better to make the review of literature more pertinent to online English teaching. In the present form, this paper focuses too generally on online teaching as a whole. Thus it is advisable for the authors to make a very brief review of general online teaching but to concentrate more on English teaching, just as the authors claim in the paper that online teaching is carried out differently to cater for the need of different subjects or disciplines.
