# Supplementary material for: Online college English education in Wuhan against the COVID-19 pandemic: Student and teacher readiness, challenges and implications
Source: PLoS One. 2021 Oct 1;16(10):e0258137. doi: 10.1371/journal.pone.0258137 (PMC8486088; doi:10.1371/journal.pone.0258137)
Supplement: Attachment — Submitted filename: Response to Reviewers.docx [file pone.0258137.s012.docx]

Response to Reviewer #1

**Thank you for allowing me to review your manuscript again. This time the authors have exerted many efforts to enhance their manuscript. Indeed, the manuscript in this version is better than that in the previous version. Since the manuscript has presented major revisions in terms of the overall arrangement, the authors can also make further improvements to make the manuscript clearer and more persuasive.**

Thank you very much for reviewing our manuscript again and recognition of our efforts in improving the manuscript. The improvements couldn’t have been made without your suggestions and those from other reviewers. We are grateful for your further suggestions and the problems are addressed as follows.

**First, the authors can further emphasize the central part of “readiness”. Since this concept is the keyword, the authors can highlight the significance of “readiness” in online EFL.**

Relevant studies were summarized to highlight the significance of “readiness” in online EFL in Section 2.2:

Several studies concluded the significance of e-readiness in online learning from different perspectives. Moftakhari [32] claimed the success of online learning entirely relied on learners’ and teachers’ readiness levels, which seems to be absolute. Piskurich [33] believed low readiness level was the main reason for failure in online learning. Students’ e-learning readiness was proved statistically as a significant predictor of their satisfaction for online instruction [34-36]. Therefore, assessing student readiness for online learning is highly relevant prior to delivering a course online either fully or hybridly and promoting student readiness is essential for successful online learning experiences [37].

**Besides, the authors can further stress their contribution to technological development and pedagogical practices to demonstrate the practicality of this manuscript. The potential readers can also grasp the persuasive information concerned with the target field.**

Implications for teaching staff, institutions and IT companies were listed in Section 5.2. Upon your advice, we checked the implications to see whether they corresponded with the findings and made some improvements.

**At last, to guarantee a more explicit manuscript structure, the authors can answer the research questions or hypotheses in the Conclusion section. Thus, this manuscript can tell the readers what have been done to investigate particular questions.**

The conclusion part was rewritten to answer the research questions briefly in order to achieve a more explicit manuscript. Detailed explanations were included in Sections 4.2-4.4 and Section 5.1, so the conclusion part was kept concise.

Response to Reviewer #5

**The author did improve the quality and the readability, however, I'm still not confident to recommend this manuscript to be published in PLOS ONE, the most important reason is that it's no longer the right time to publish articles related to people's readiness and challenges during the COVID-19, since it's already the post-pandemic era now.**

Thank you for your favorable comments on our revised version. We were aware of the timeliness of the submission, but it took us much longer time to analyze the data and write the draft than expected. Unfortunately, the pandemic still persists in many parts of the world, though it’s already the post-pandemic era and people have been trying to live with the virus. Here in China, classes were moved online occasionally in several cities when local cases were confirmed, and many Chinese students who were enrolled in international programs were having remote classes due to restrictions on international travel. In addition, though the research was conducted during the pandemic and the analysis focused on people’s readiness and challenges during that period, the results can provide some implications for online English teaching in general as indicated in the manuscript. Therefore, we sincerely hope to get an opportunity to publish the research. Moreover, thanks for your inspiration, we may explore more issues in online language teaching in the post-pandemic era.

Response to Reviewer #6

**1. In the abstract, the sentence "Technical barriers should be removed, readiness evaluation and instructor training are also necessary." seems ungrammatical. It needs to be further polished.**

Thank you for carefully reviewing our manuscript again. The sentence was ambiguous and it was rewritten as: They are supposed to remove technical barriers for teachers and students, and assess the readiness levels of both cohorts before launching English courses online. Institutions should also arrange proper training for instructors involved, especially about pedagogical issues.

The language of the manuscript was polished again upon suggestions from you and another reviewer.

**2. In addition, the abstract starts with the method part, which is OK. But I think it is better to give some background introduction at the very beginning of the abstract to illustrate the research domain.**

Thanks for your advice and we take it. The following sentence was added at the beginning of the abstract:

Online education, including college English education, has been developing rapidly in the recent decade in China. Such aspects as e-readiness, benefits and challenges of online education were well-researched under normal situations, but fully online language teaching on a large-scale in emergencies may tell a different story.

Response to Reviewer #8

**The authors have well addressed the concerns of the reviewers, and I very much appreciate what the authors have done in the revision.**

**With that said, I would suggest the authors closely read the manuscript again, although the language has very much improved compared with that of the last version. One example lies in Line 55, p.4, where "at a short notice" seemingly should be "at short notice" (see https://www.collinsdictionary.com/dictionary/english/at-short-notice and https://www.ldoceonline.com/dictionary/at-short-notice).**

Thank you for reviewing our manuscript again and giving us positive comments on our last revision. We are so grateful because the manuscript couldn’t have been improved without constructive suggestions from you and other reviewers.

We feel ashamed of our silly mistakes (like the one you pointed out), and the language was polished again. Hopefully it has been improved. We are truly grateful for and inspired by your meticulousness.

Response to Journal Requirements

**Please review your reference list to ensure that it is complete and correct. If you have cited papers that have been retracted, please include the rationale for doing so in the manuscript text, or remove these references and replace them with relevant current references. Any changes to the reference list should be mentioned in the rebuttal letter that accompanies your revised manuscript. If you need to cite a retracted article, indicate the article’s retracted status in the References list and also include a citation and full reference for the retraction notice.**

The reference list was checked again and no retracted paper was cited. However, when revising the manuscript, two new references were added and the order of several references was changed. The reference list of this revised version was complete and correct.
